# Abdominal functional electrical stimulation to assist ventilator weaning in critical illness: a double-blinded, randomised, sham-controlled pilot study

**DOI:** 10.1186/s13054-019-2544-0

**Published:** 2019-07-24

**Authors:** Euan J. McCaughey, Annemijn H. Jonkman, Claire L. Boswell-Ruys, Rachel A. McBain, Elizabeth A. Bye, Anna L. Hudson, David W. Collins, Leo M. A. Heunks, Angus J. McLachlan, Simon C. Gandevia, Jane E. Butler

**Affiliations:** 1 Neuroscience Research Australia, 139 Barker Street, Randwick, NSW 2031 Australia; 2 School of Medical Sciences, University of New South Wales, Kensington, NSW 2052 Australia; 3 Department of Intensive Care Medicine, Amsterdam UMC, Vrije Universiteit Amsterdam, De Boelelaan, 1117 Amsterdam, The Netherlands; 4 Prince of Wales Hospital, Randwick, NSW 2031 Australia; 5 Liberate Medical LLC, 6400 Westwind Way, Suite A, Crestwood, KY 40014 USA

**Keywords:** Critical illness, Electrical stimulation, Mechanical ventilation, Respiratory function, Respiratory muscles

## Abstract

**Background:**

For every day a person is dependent on mechanical ventilation, respiratory and cardiac complications increase, quality of life decreases and costs increase by > $USD 1500. Interventions that improve respiratory muscle function during mechanical ventilation can reduce ventilation duration. The aim of this pilot study was to assess the feasibility of employing an abdominal functional electrical stimulation (abdominal FES) training program with critically ill mechanically ventilated patients. We also investigated the effect of abdominal FES on respiratory muscle atrophy, mechanical ventilation duration and intensive care unit (ICU) length of stay.

**Methods:**

Twenty critically ill mechanically ventilated participants were recruited over a 6-month period from one metropolitan teaching hospital. They were randomly assigned to receive active or sham (control) abdominal FES for 30 min, twice per day, 5 days per week, until ICU discharge. Feasibility was assessed through participant compliance to stimulation sessions. Abdominal and diaphragm muscle thickness were measured using ultrasound 3 times in the first week, and weekly thereafter by a blinded assessor. Respiratory function was recorded when the participant could first breathe independently and at ICU discharge, with ventilation duration and ICU length of stay also recorded at ICU discharge by a blinded assessor.

**Results:**

Fourteen of 20 participants survived to ICU discharge (8, intervention; 6, control). One control was transferred before extubation, while one withdrew consent and one was withdrawn for staff safety after extubation. Median compliance to stimulation sessions was 92.1% (IQR 5.77%) in the intervention group, and 97.2% (IQR 7.40%) in the control group (*p* = 0.384). While this pilot study is not adequately powered to make an accurate statistical conclusion, there appeared to be no between-group thickness changes of the rectus abdominis (*p* = 0.099 at day 3), diaphragm (*p* = 0.652 at day 3) or combined lateral abdominal muscles (*p* = 0.074 at day 3). However, ICU length of stay (*p* = 0.011) and ventilation duration (*p* = 0.039) appeared to be shorter in the intervention compared to the control group.

**Conclusions:**

Our compliance rates demonstrate the feasibility of using abdominal FES with critically ill mechanically ventilated patients. While abdominal FES did not lead to differences in abdominal muscle or diaphragm thickness, it may be an effective method to reduce ventilation duration and ICU length of stay in this patient group. A fully powered study into this effect is warranted.

**Trial registration:**

The Australian New Zealand Clinical Trials Registry, ACTRN12617001180303. Registered 9 August 2017.

## Background

Approximately 33% of critically ill patients treated in intensive care units (ICUs) require mechanical ventilation to support respiration, some for a few hours, and others for months [1]. During this time, disuse atrophy of the major respiratory muscles, namely the diaphragm, abdominal and intercostal muscles, may occur [2]. This reduces respiratory function and leads to a range of complications including difficulty weaning from mechanical ventilation [3], increased mortality, respiratory and cardiac complications, readmissions to hospital and intensive care [2–6], and decreased quality of life [7, 8]. While a lifesaving intervention, need for mechanical ventilation is also associated with additional health care costs [9]. Interventions that reduce respiratory muscle atrophy or increase respiratory muscle strength are likely to reduce mechanical ventilation duration, with a direct impact on morbidity and mortality, quality of life and costs to the health care provider.

Functional electrical stimulation (FES) is the application of a train of electrical pulses to a motor nerve, causing the associated muscle to contract. Transcutaneous FES of the abdominal muscles, termed abdominal FES, can improve respiratory function [10–13] and assist ventilator weaning in spinal cord injury [12, 14]. Unlike inspiratory muscle training, which has been shown to improve weaning outcomes for difficult to wean patients [15, 16], abdominal FES does not require patient participation or cooperation [16]. A pilot study of 25 ventilated critically ill participants showed that FES of the rectus abdominis and pectoral muscles maintained respiratory muscle thickness to a greater degree than sham stimulation and shortened ICU length of stay [17]. This is despite the rectus abdominis muscles making minimal contribution to expiratory pressures [18, 19], and that stimulation was not applied in synchrony with respiration (increasing the risk of patient-ventilator asynchrony and increasing the load of breathing). FES of muscles in the upper legs of ventilated critically ill patients has also been shown to reduce ventilation duration [20]. Although widely advocated as an effective technique to maintain muscle mass and reduce critical illness polyneuromyopathy for critically ill patients [21, 22], it does not directly target the respiratory muscles. As the abdominal muscles play an active role in cough generation and respiration during respiratory distress [23] and we have previously shown that abdominal FES is an effective way to improve cough function [13], abdominal FES may provide a more direct, practical and efficacious way to reduce mechanical ventilation duration in critical illness. This hypothesis is further supported by the fact that respiratory muscle strength, as measured by maximum expiratory pressure (MEP) and cough peak flow (CPF), has been shown to be an independent predictor of delayed extubation, weaning success, morbidity and mortality [24, 25]. The primary aim of this pilot study was to assess the feasibility of employing an abdominal FES training program with critically ill mechanically ventilated patients. Secondary objectives were to investigate the effect of abdominal FES on muscle atrophy, mechanical ventilation duration and ICU length of stay. The data collected from this study will be used to assess feasibility and estimate sample size, for a fully powered study to ascertain whether abdominal FES can reduce mechanical ventilation in critical illness.

## Methods

### Study design

A double-blinded, randomised, sham-controlled pilot study was conducted in the 12 bed ICU of a metropolitan teaching hospital. The study was approved by the local research ethics board.

The aim of this pilot study was to assess the feasibility of employing an abdominal FES training program with critically ill mechanically ventilated patients. Secondary objectives were to investigate whether abdominal FES affects abdominal muscle and diaphragm thickness, respiratory function, ventilation duration, ICU length of stay and mortality in this population. Dall’ Acqua et al. [17] found a medium effect (effect size = 0.75) from abdominal FES on abdominal muscle thickness. Our study improves on this method by stimulating the posterolateral abdominal wall as opposed to the rectus abdominis muscles and applying stimulation in synchrony with respiration. Assuming our intervention will also have a medium effect size on abdominal muscle thickness, the optimal sample size for this pilot study is 10 participants per arm [26].

### Participants

**Fig. 1:**
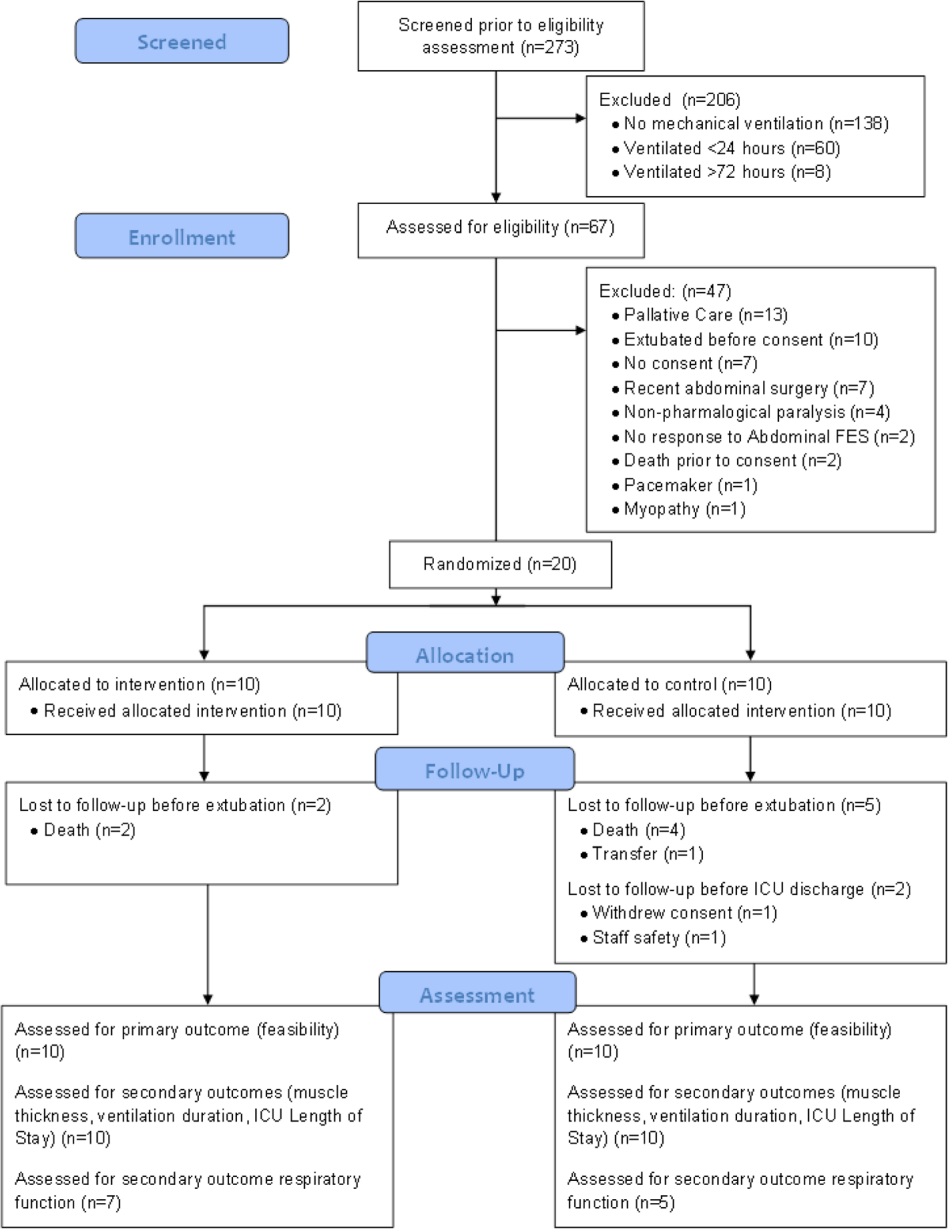
Consort flow diagram of patients admitted to the intensive care unit (ICU) and the randomisation process

All consecutive admissions (*n* = 273) between 1 November 2017 and the 12 April 2018 were screened against the eligibility criteria (Fig. 1). Patients were eligible if they were ≥ 18 years of age and dependent on mechanical ventilation due to critical illness. Patients were excluded if they were expected to be ventilated for < 24 h or already ventilated for > 72 h, were pregnant, had non-pharmacological paralysis (e.g. spinal cord injury), had physical obstacles that prevent abdominal FES (e.g. abdominal trauma, pacemaker), had a diagnosed terminal illness, had no response to abdominal FES (e.g. lower motor neuron impairment or obese) or had abdominal surgery within 4 weeks prior to potential inclusion. Similar criteria were used in a previous trial of the effectiveness of FES of the quadriceps to reduce critical illness polyneuromyopathy in critically ill mechanically ventilated patients [22].

### Stimulation

Twenty participants were randomised to receive active (intervention) or sham (control) abdominal FES (Table 1). Participants received the first session of their allocated intervention ~ 48 h post initiation of mechanical ventilation (enabling washout of neuromuscular blocking agents). The set up for both groups was identical, with the only difference being the stimulation parameters. Stimulation was applied for 30 min, twice per day, 5 days per week (including first 5 days consecutively), until discharge from the ICU, via surface electrodes (5 cm × 10 cm rectangular, UF2040, Axelgaard, USA). Electrodes were placed posteriorlaterally over the abdominal wall designed to activate the transversus abdominis and internal and external oblique muscles as previously described [18]. Stimulation was applied during exhalation using a commercially available abdominal FES device (Empi Continuum, Empi Inc., USA) with automatic synchronisation with the participant’s breathing achieved using an investigational device (VentFree VF03-K, Liberate Medical LLC, USA, note not approved for therapeutic use) connected between the y-piece of the mechanical ventilator and the endotracheal tube. The active group received abdominal FES at an intensity that caused a strong visible muscle contraction (median 60 mA [range 50–65 mA]), with a frequency of 30 Hz and a pulsewidth of 350 μs. The stimulation current in the control group was set at 10 mA (possible sensation but no muscle contraction), with a frequency of 10 Hz and a pulsewidth of 350 μs. Similar training protocols have been used in other studies performed by the research team [11, 12, 14].

**Table 1:** Participant information. All ventilator settings refer to first day of study. APACHE III score was calculated in the first 24 h of ICU admission as described by Knaus et al. [27]. *PEEP* positive end-expiratory pressure, *FiO*_*2*_ fraction of inspired oxygen, *IQR* interquartile range

	Active (*n* = 10)	Control (*n* = 10)
Age (years) [median (IQR)]	56.5 (18.50)	61.0 (17.25)
Gender *[M/F]*	7/3	5/5
Severity of illness at ICU admission		
APACHE III score [median (IQR)]	81.5 (37.75)	82.0 (14.00)
Diagnostic category at admission [*n* (%)]		
Brain injury	6 (60%)	2 (20%)
Sepsis/septic shock	0	3 (30%)
Respiratory failure	0	2 (20%)
Trauma	0	0
Post-surgical	1 (10%)	0
Meningitis	1 (10%)	1 (10%)
Other	2 (20%)	2 (20%)
Baseline ventilation characteristics		
Mode of ventilation [*n* (%)]		
Synchronized intermittent-mandatory ventilation	10 (100%)	9 (90%)
Adaptive pressure ventilation	0	1 (10%)
PEEP (cmH_2_O) [median (IQR)]	10.0 (3.50)	10.0 (2.25)
FiO_2_ (%) [median (IQR)]	25.0 (6.75)	30.0 (10.00)
Exposure to intervention (min) [median (IQR)]	366 (293.8)	555 (492.5)

To achieve blinding, the researcher administering abdominal FES drew each participant’s bedside curtain while preparing the device. This person did not perform any outcome measurements. The machine was covered with a towel or sheet, and the participants’ abdomen covered with a bed sheet so that participants, family members and caregivers could not see the machine or whether stimulation resulted in muscle contractions. Outcome assessors were never in the room when stimulation was delivered. Although participants were not informed of their randomisation allocation, they could notice the contractions caused by abdominal FES (compared to the control) and therefore could become aware of the allocation. Participants were instructed not to discuss their perception of allocation with outcome assessors, other participants or clinical staff.

### Data collection

Ultrasound was performed at the end of exhalation (without stimulation) to measure the thickness of the rectus abdominis, internal and external oblique and transversus abdominis muscles and diaphragm before the first abdominal FES session, twice more in the first week of participation, and then weekly until ICU discharge. All measurements were taken from muscles on the right-hand side of the participant by the same assessor at all assessment sessions. To measure the rectus abdominis, the probe was firstly placed on the midline of the abdomen, 2 cm above the umbilicus to identify the linea alba. The probe was then moved laterally until the right rectus abdominis muscle became visible, with the probe then moved in the cranial and caudal directions until the maximum thickness of the muscle was identified. From the position of the rectus abdominis, the probe was moved to the right until the lateral abdominal muscles became visible, and moved laterally until the upper and lower limits of each muscle were parallel to each other. This was approximately at the anterior axillary line. Minimal pressure to the skin was applied during these measurements to limit muscle deformation. For the diaphragm, the probe was placed parallel to the anterior axillary line in the intercostal space between the 9th and 10th rib and moved in the cranial and caudal directions until the pleural line was identified. From this point, the probe was moved approximately 1 or 2 intercostal spaces lower to identify the costal diaphragm in the zone of apposition. In all measurements the, probe was placed perpendicular to the skin.

Respiratory function was measured via forced vital capacity (FVC), forced expiratory volume in 1 s (FEV_1_), peak expiratory flow (PEF), maximum inspiratory pressure (MIP) and maximum expiratory pressure (MEP), as soon as possible after the participant was able to breathe independently. FVC, FEV_1_ and PEF were measured with a handheld spirometer (One Flow FVC Memo, Clement Clarke International, UK) by asking the participant to exhale as fully and as forcefully as possible (verbal encouragement provided) from total lung capacity. MIP and MEP were measured using a hand-held pressure meter (MicroRPM, Vyaire Medical, USA), with participants inhaling and exhaling as fully and as forcefully as possible (verbal encouragement provided) against an occluded airway from residual volume and total lung capacity, respectively. The size of the filter approved for use with our mouth pressure device was not compatible with the tracheostomies being used at the study site. As such, MIP and MEP were not recorded from patients with tracheostomies. All measurements were recorded with the participant supine. When possible, each measurement was repeated until three reproducible results within 5% were registered, and the greatest value used for analysis [28].

Ventilation duration (defined as the total number of days from the onset of ventilation until the first successful extubation of more than 48 h during ICU stay [22]) and ICU length of stay (the number of days from ICU admission to ICU discharge) were obtained via chart review by a blinded assessor at ICU discharge, while mortality was obtained from the participants’ medical record by the same blinded assessor 6 weeks post ICU discharge. Participants without a tracheostomy were extubated by progressively reducing ventilator support. Here, ventilator rate, pressure support, and positive end-expiratory pressure (PEEP) were decreased while respiratory rate, respiratory effort, tidal volume and blood gases were monitored. When support reached low levels, typically PEEP and pressure support of 8 and 7 cmH_2_O, respectively, participants were extubated based on clinical judgement. Participants with a tracheostomy were weaned from ventilatory support via a similar scheme and then progressive ventilator-free breathing.

### Analysis

Categorical data are summarised in terms of the number of participants with data at the relevant time point (*n*) and as a percentage of all participants. Continuous data are expressed as median and interquartile range (IQR), or mean and standard deviation unless otherwise stated. Compliance is the number of sessions completed as a percentage of all sessions that should have been completed between randomisation and completion or withdrawal and is considered a continuous variable. In cases where participants underwent a double session (i.e. one 60-min session instead of two 30-min sessions), this was regarded as compliant and taken as two completed sessions for analysis purposes. A Mann-Whitney *U* test was used to compare compliance between groups. The combined thickness of the internal and external oblique and transversus abdominis muscles (i.e. from the upper fascia of the external oblique to the lower fascia of the transversus abdominis) was also analysed to increase accuracy, with this measurement referred to here as the combined lateral abdominal muscles. The mean of at least three ultrasound images for each muscle group at each assessment session was used for analysis. Blinded researchers in another country also checked the marked images to verify correct muscle identification and marker placement. In cases of disagreement, where the fascia was not clear in saved images, or the correct muscle group was not obvious, the data were excluded (18.2% [*n* = 14], 27.3% [*n* = 21], and 22.1% [*n* = 17] of sessions were excluded for the rectus abdominis, combined lateral abdominal muscles and diaphragm, respectively). Change from baseline of rectus abdominis, combined lateral abdominal, internal and external oblique and transversus abdominis muscles and diaphragm thickness were treated as continuous variables and analysed using a linear mixed effects model with fixed factors of baseline thickness, treatment, assessment session and treatment by assessment session interaction, and a random effect of participant (change from baseline thickness ~ baseline thickness + treatment + assessment session + (treatment * assessment session) + (1|patient)) [29]. These mixed models compare the thickness of the muscles over time. Due to the small sample size, and the risk of a normality test being underpowered, we followed the statistical methods in Dall’ Acqua et al. [17], where the distribution of muscle thickness was assumed normal.

There are no respiratory function measures for the participants who died during the study. The Mann-Whitney *U* test, as a distribution-free non-parametric test, was used to analyse respiratory function data. Ventilation duration and ICU length of stay were analysed using Gray’s test in the survival analysis [30], with the competing risks of death or withdrawal of treatment (e.g. ventilator support) with the intention of subsequent death. Gray’s test compares cause-specific cumulative incidence curves. In the case where less than 50% of participants achieved the outcome, due to either competing events or censoring, the median time to the outcome was not estimable. The sample size required for a larger study was also calculated based on survival test and cause-specific hazard approach accounting for competing events. All analyses were performed using SPSS (Version 22, IBM Corp, NY, USA).

## Results

### Study population and compliance

Twelve males and eight females, with a median age of 56.5 years in the active group and 61.0 years in the control group, were recruited for this study (Table 1). Fourteen patients survived to ICU discharge (8 active, 6 control). One control participant was transferred to another hospital before extubation. After extubation, one further control participant withdrew consent and one was withdrawn due to violent behaviour. The median time on mechanical ventilation before starting the intervention was 1.5 days (IQR 1 day). The median time on the study for participants who died was 11.5 days (IQR 13.25 days). Median compliance for the training sessions was 92.1% (IQR 5.77%) in the active group and 97.2% (IQR 7.40%) in the control group (*p* = 0.384), with active participants having a median exposure to the intervention of 366 min (IQR 293.8 min) and control participants 555 min (IQR 492.5 min) (Table 1).

### Adverse events

There were eight non-serious adverse events in the active group and 14 in the control group and two serious adverse events (death) in the active group and eight in the control group (four death, three cardiac events, one re-intubation) (Table 2). Only one participant, who was a control, suffered multiple serious adverse events (2 cardiac events and death). For adverse events, two active participants experienced three events (2 hospital-acquired infections and pneumonia; 2 hospital-acquired infections and tracheostomy), and one had one adverse event and a serious adverse event (blocked pressure drain and death). In the control group, one participant experienced four adverse events (2 hospital-acquired infections, pneumonia and tracheostomy), two had three adverse events and a serious adverse event (high respiratory rate, hospital-acquired infection, diarrhoea and serious cardiac event; 2 hospital-acquired infections, laparotomy and reintubation) and one experienced an adverse event and serious adverse event (poor wound healing and death). All other adverse or serious adverse events were experienced by individual participants. An independent safety data monitoring committee judged that none of the serious adverse events were related to the intervention.

**Table 2:** Adverse events stratified by group. Serious adverse events and non-serious adverse events are reported separately. All data are reported as absolute number of events, as well as the proportion of all serious adverse or non-serious adverse events per group. While some participants experienced multiple adverse events (see the “Results” section: “Adverse events”), none of the adverse events reported here were deemed to be related to the intervention

	Active	Control
Serious adverse events		
Death	2 (100%)	4 (50%)
Serious cardiac events	0	3 (37.5%)
Respiratory failure requiring reintubation	0	1 (12.5%)
Total	2	8
Non-serious adverse events, *n* (%)		
Hospital-acquired infection	4 (50%)	6 (42.9%)
Tracheostomy	1 (12.5%)	1 (7.1%)
Blocked intracranial pressure drain	1 (12.5%)	0
Headache	1 (12.5%)	0
Pneumonia	1 (12.5%)	0
Diarrhoea	0	1 (7.1%)
Gout	0	1 (7.1%)
High respiratory rate	0	1 (7.1%)
Laparotomy	0	1 (7.1%)
Cardiac event	0	1 (7.1%)
Poorly healing surgical wound	0	1 (7.1%)
Violence to staff	0	1 (7.1%)
Total	8	14

### Muscle atrophy

There was no difference in the longitudinal changes from baseline in the thickness of rectus abdominis, diaphragm and combined lateral abdominal muscles between groups at any assessment sessions (Table 3). Further analyses were also conducted on each individual muscle within the combined lateral abdominal muscles, where there did appear to be a change in the thickness of the transversus abdominis at day 3 (0.85 vs. − 0.12, *p* = 0.032). It should be noted that the results for assessment sessions beyond day 5 were not stable or not estimable due to the small or no sample sizes.

**Table 3:** Group comparison of change from baseline in thicknesses of rectus abdominis, diaphragm, combined lateral abdominal muscle and each individual muscle by assessment session. Data are summarised as mean ± SD (*n*). Active versus control analysed using a least square mean difference based on a mixed effects model for repeated measures. *p* values based on a mixed effects model for repeated measures. *NE* not estimable

Assessment session	Active	Control	Active–control	*p* value
Change from baseline in rectus abdominis thickness (mm) by assessment session				
Day 3	0.33 ± 0.909 (6)	− 0.10 ± 0.451 (9)	0.61 (− 0.13, 1.35)	0.099
Day 5	− 0.03 ± 0.871 (5)	0.29 ± 0.759 (6)	− 0.11 (− 0.96, 0.74)	0.785
Day 12	0.68 ± 0.165 (2)	0.35 ± 0.453 (4)	0.09 (−1.07, 1.25)	0.877
Day 19	NE	1.22 ± 1.167 (2)	NE	NE
Day 26	NE	1.37 ± 0.813 (2)	NE	NE
Day 33	NE	2.90 ± 0.071 (2)	NE	NE
Change from baseline in diaphragm thickness (mm) by assessment session				
Day 3	− 0.17 ± 0.274 (7)	− 0.18 ± 0.207 (6)	0.06 (− 0.23, 0.36)	0.652
Day 5	− 0.11 ± 0.404 (7)	− 0.18 ± 0.225 (4)	− 0.04 (− 0.38, 0.30)	0.794
Day 12	− 0.13 ± 0.305 (4)	− 0.03 ± 0.336 (4)	− 0.07 (− 0.44, 0.31)	0.698
Day 19	NE	0.30 (1)	NE	NE
Day 26	NE	− 0.72 (1)	NE	NE
Day 33	NE	0.08 (1)	NE	NE
Change from baseline in combined lateral abdominal muscle thickness (mm) by assessment session				
Day 3	2.51 ± 2.535 (4)	− 0.01 ± 2.113 (9)	3.05 (− 0.35, 6.44)	0.074
Day 5	0.63 ± 0.701 (4)	0.28 ± 2.186 (7)	1.23 (− 2.29, 4.75)	0.463
Day 12	1.55 (1)	− 0.88 ± 2.902 (5)	3.66 (− 1.96, 9.28)	0.183
Day 19	NE	0.22 ± 3.005 (2)	NE	NE
Day 26	NE	4.12 ± 4.419 (2)	NE	NE
Day 33	NE	3.75 (1)	NE	NE
Change from baseline in external oblique thickness (mm) by assessment session				
Day 3	0.52 ± 0.685 (4)	− 0.05 ± 1.153 (9)	1.08 (− 0.07, 2.23)	0.064
Day 5	0.09 ± 0.954 (4)	− 0.35 ± 0.881 (7)	0.95 (− 0.24, 2.13)	0.108
Day 12	− 1.14 (1)	− 0.49 ± 0.248 (5)	− 0.68 (− 2.57, 1.20)	0.450
Day 19	NE	0.35 ± 0.071 (2)	NE	NE
Day 26	NE	0.77 ± 0.177 (2)	NE	NE
Day 33	NE	2.20 (1)	NE	NE
Change from baseline in internal oblique thickness (mm) by assessment session				
Day 3	0.79 ± 1.718 (4)	0.13 ± 0.936 (9)	1.08 (− 1.24, 3.40)	0.335
Day 5	− 0.43 ± 1.040 (4)	0.58 ± 1.694 (7)	− 0.37 (− 2.74, 2.01)	0.746
Day 12	0.09 (1)	− 0.05 ± 2.385 (5)	1.61 (− 2.16, 5.39)	0.375
Day 19	NE	0.50 ± 1.838 (2)	NE	NE
Day 26	NE	2.33 ± 2.722 (2)	NE	NE
Day 33	NE	1.75 (1)	NE	NE
Change from baseline in transversus abdominis thickness (mm) by assessment session				
Day 3	0.85 ± 1.065 (4)	− 0.12 ± 0.849 (9)	1.04 (0.10, 1.98)	0.032
Day 5	0.56 ± 0.775 (4)	0.10 ± 0.532 (7)	0.68 (− 0.32, 1.68)	0.168
Day 12	1.74 (1)	− 0.11 ± 0.318 (5)	2.28 (0.50, 4.06)	0.016
Day 19	NE	− 0.18 ± 0.742 (2)	NE	NE
Day 26	NE	0.78 ± 1.025 (2)	NE	NE
Day 33	NE	0.35 (1)	NE	NE

### Respiratory function

Respiratory function was assessed at a median of 6 days (IQR 3 days) from randomisation for the active group and 15 days (IQR 15 days) from randomisation for the control group (*p* = 0.084). In the active group, one participant was unable to perform all respiratory function measures due to delirium and one was unable to adequately perform MIP and MEP measurements due to tracheostomy. In the control group, one participant was unable to perform all respiratory measures due to transfer to another hospital and one was unable to perform MIP and MEP due to tracheostomy. There was no difference in FVC (*p* = 0.371), FEV_1_ (*p* = 0.371), MIP (*p* = 0.762), MEP (*p* = 0.283) or PEF (*p* = 0.061) between groups (Table 4).

**Table 4:** Respiratory function. Respiratory function is analysed as soon as possible after the participant is able to breathe independently. There are no respiratory function measures for the six participants who died during the study. See the section “Analysis” for further information relating to who participated in respiratory function measurements. Analysis was performed using the Mann-Whitney *U* test. All data are shown as: Median (interquartile range (IQR)) [number of participants providing data (*N*)]. *MIP* maximum inspiratory pressure, *MEP* maximum expiratory pressure, *PEF* peak expiratory flow, *FVC* forced vital capacity, *FEV*_*1*_ forced exhaled volume in 1 s

	Active (median (IQR) [*N*])	Control (median (IQR) [*N*])	*p* value
MIP (cmH_2_O)	29.0 (26.75) [*N* = 6]	32.5 (9.50) [*N* = 4]	0.762
MEP (cmH_2_O)	35.5 (12.75) [*N* = 6]	26.0 (4.00) [*N* = 4]	0.283
PEF (L/min)	127.5 (62.5) [*N* = 7]	50.0 (55.00) [*N* = 5]	0.061
FVC (L)	1.3 (0.58) [*N* = 7]	0.9 (0.90) [*N* = 5]	0.371
FEV_1_ (L)	0.9 (0.48) [*N* = 7]	0.6 (0.70) [*N* = 5]	0.371

### Clinical outcomes

**Fig. 2:**
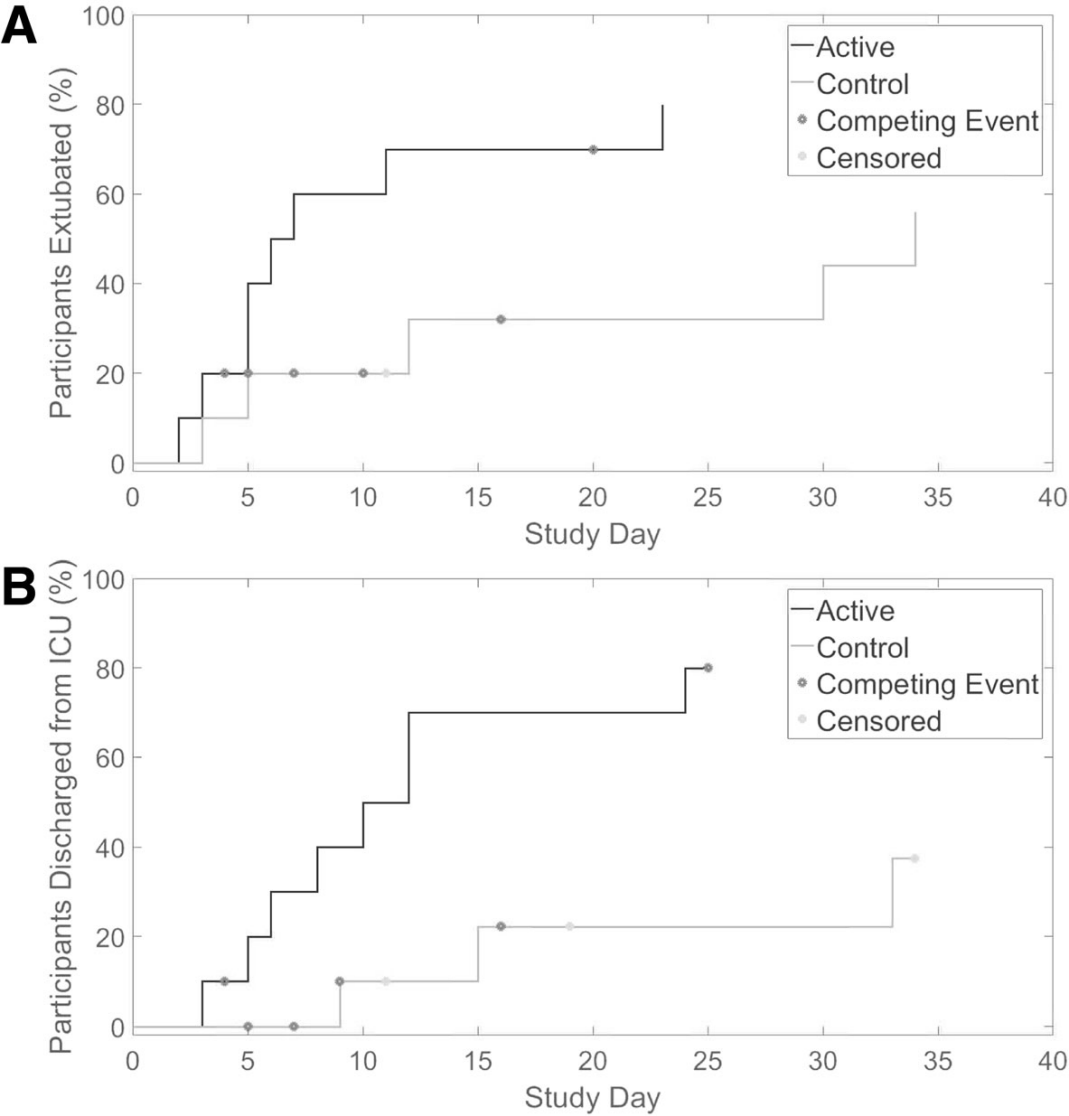
Cumulative incidence curves for mechanical ventilation duration (**a**) and intensive care unit (ICU) length of stay (**b**). Fourteen participants survived to ICU discharge (8 active (dark grey), 6 control (light grey)). One control participant was transferred to another hospital before extubation and as such was censored from both the ventilation duration and ICU length of stay analysis. After extubation, one control participant withdrew consent and one was withdrawn due to threatening behaviour, both were censored from the ICU length of stay analysis. Competing events were death or withdrawal of treatment (e.g., ventilator support) with the intention of subsequent death (marked with a dark grey asterisk). Participants who were censored are represented by a light grey asterisk. *ICU* intensive care unit

Ventilation duration (median 6.5 versus 34 days, Gray’s test *p* = 0.039) and ICU length of stay (median 11 versus not estimable days, Gray’s test *p* = 0.011) were shorter in the active compared to the control group (Fig. 2). Of the 13 participants liberated from mechanical ventilation, nine (69.2%, 6 active, 3 control) were liberated by reducing ventilator support. The four remaining participants (two active, two control) were extubated via progressive ventilator-free breathing. The median time from initiation of progressive ventilator-free breathing to extubation was 9.5 days (IQR 7.75 days).

There was no difference in mortality between groups (*p* = 0.629).

## Discussion

The aim of this pilot study was to assess the feasibility of employing an abdominal FES training program with critically ill mechanically ventilated patients. We also investigated the effect of abdominal FES on respiratory muscle atrophy, mechanical ventilation duration and ICU length of stay. Our compliance to the stimulation sessions of > 90% in both groups demonstrates the feasibility of applying this intervention in the critically ill mechanically ventilated population. While this pilot study is not adequately powered to make an accurate statistical conclusion, we did not find a longitudinal difference in respiratory muscle thickness between groups. However, ICU length of stay and duration of mechanical ventilation duration were shorter in the abdominal FES than the control group. This provides justification for a fully powered study to determine whether abdominal FES can reduce mechanical ventilation in critical illness. Such a study would require 254 participants (based on a cause-specific hazard approach accounting for competing events, assuming 60% of intervention and 45% of controls being liberated from the ventilator by day 9, logrank test (2 sided), *α* = 0.05 (two sided), *β* = 0.1, mortality at day 9 = 20%, 10% loss to follow-up).

Routsi et al. [22] demonstrated that participants who received FES of the quadriceps had a ventilation duration of 7 days compared to 10 days for controls (*p* = 0.07). Abu-Khaber et al. [20] found the same technique reduced ventilation duration from 12 to 9 days in a similar group of patients (*p* = 0.048). However, while advocated clinically as a way to reduce ventilation duration [21, 22], this technique does not directly target the respiratory muscles. Here, we found abdominal FES appeared to reduce ventilation duration and ICU length of stay. In agreement, Dall’Acqua et al. [17] found that FES of the rectus abdominis and intercostal muscles reduced ICU length of stay (*p* = 0.045). In contrast, Routsi et al. [22] found that FES of the quadriceps did not change ICU length of stay (*p* = 0.11). With each ICU bed day in Australian public hospitals estimated at $A6141 (compared to $A2351 for a general ward bed) [31], a reduction in ICU length of stay would result in a significant cost saving for local health care providers. As such, abdominal FES may offer a useful clinical addition or alternative to FES of the quadriceps and is worthy of further exploration.

Our finding that there appeared to be no longitudinal change in the thickness of the rectus abdominis muscles in either the intervention or control group is in contrast to Dall’Acqua et al. [17], who found no change in rectus abdominis thickness in patients who received FES of the rectus abdominis and intercostal muscles, but a significant 16.3% decrease in the control group. This, coupled with the fact that we did not observe a difference in diaphragm thickness between the groups, may indicate that the mechanisms of abdominal FES to reduce ventilation duration are not solely based on muscle thickness. Furthermore, MIP and MEP are good indicators of respiratory muscle strength. [32] While these outcomes and those of lung function were not different between the two groups, a previous systematic review has shown that abdominal FES can improve respiratory function in spinal cord injury [13]. Further study of the effect of abdominal FES on respiratory function in this population is warranted.

Our average recruitment rate of 4 participants per month was higher than expected and shows the feasibility of a larger study, particularly if it were multi-institutional. However, 12.6% of all interventions in this study were double sessions (stimulation applied for 1 consecutive hour, as opposed to two 30-min sessions), largely due to staffing issues and difficulty accessing participants (e.g. they were away for a procedure). This suggests that one training session per day may be more practical for a follow-up study. The mortality rate in this study (30%) was slightly lower than that in a larger study by Routsi et al. (35%) that employed the same inclusion and exclusion criteria [22], but is in line with large epidemiological studies of ICU patients [33].

### Limitations

While ultrasound has been shown to be a reliable measure of diaphragm thickness in the ICU [34], ultrasound measurements of the abdominal muscles and diaphragm recorded here had large intra- and inter-participant variability. This may have been due, at least in part, to a number of these critically ill patients having fluid overload, large amounts of oedema and high intra-abdominal pressures, or a combination of all three. This could have led to changes in muscle architecture unrelated to atrophy or abdominal FES. However, it should be noted that fluid imbalance alone has been shown not to affect diaphragm thickness [34, 35]. Difficulty with the ultrasound measurements led to a number of sessions having to be excluded from the analysis. As a result, more robust methods are needed to measure respiratory muscle thickness in a large clinical trial.

The majority of the analysis in this pilot study was affected by post-randomisation events and effects, particularly death. This was only accounted for in the analysis of ventilation duration and ICU length of stay, which employed Gray’s test with death and withdrawal of treatment treated as competing events or censoring. As such, there may be some bias in the other outcome measures due to the larger number of control participants not completing the study. Analysis of a larger study will need to account for these post-randomisation events in all outcome measures.

## Conclusion

This pilot study demonstrates the feasibility of employing an abdominal FES training program with critically ill mechanically ventilated patients. While there were no longitudinal changes in respiratory muscle thickness between groups, participants who received abdominal FES had a shorter mechanical ventilation duration and ICU length of stay. A fully powered study into this effect is now warranted, with a positive outcome likely to lead to the rapid clinical translation of this technique. This should lead to reduced morbidity and mortality, improved quality of life and a significant cost saving for the health care provider.

## Data Availability

The datasets used and analysed during the current study are available from the corresponding author on reasonable request.

## Abbreviations

FES: Functional electrical stimulation
FEV_1_: Forced exhaled volume in 1 s
FiO_2_: Fraction of inspired oxygen
FVC: Forced vital capacity
ICU: Intensive care unit
IQR: Interquartile range
MEP: Maximum expiratory pressure
MIP: Maximum inspiratory pressure
PEEP: Positive end-expiratory pressure
PEF: Peak expiratory flow
